# Genetic variability assessment of 127 *Triticum turgidum* L. accessions for mycorrhizal susceptibility-related traits detection

**DOI:** 10.1038/s41598-021-92837-1

**Published:** 2021-06-28

**Authors:** Paola Ganugi, Alberto Masoni, Cristiana Sbrana, Matteo Dell’Acqua, Giacomo Pietramellara, Stefano Benedettelli, Luciano Avio

**Affiliations:** 1 Department of Agriculture, Food, Environment and Forestry (DAGRI), University of Florence, Piazzale delle Cascine, 18, 50144 Florence, FI Italy; 2 Department of Biology, University of Florence, Via Madonna del Piano, 6, 50019 Sesto Fiorentino, FI Italy; 3 Institute of Agricultural Biology and Biotechnology, CNR, Via Moruzzi 1, 56124 Pisa, Italy; 4 Sant’Anna School of Advanced Studies, Piazza Martiri Della Libertà, 33, 56127 Pisa, Italy; 5 Department of Agriculture, Food and Environment (DAFE), University of Pisa, Via del Borghetto 80, 56124 Pisa, Italy

**Keywords:** Genetics, Microbiology, Plant sciences

## Abstract

Positive effects of arbuscular mycorrhizal fungi (AMF)—wheat plant symbiosis have been well discussed by research, while the actual role of the single wheat genotype in establishing this type of association is still poorly investigated. In this work, the genetic diversity of *Triticum turgidum* wheats was exploited to detect roots susceptibility to AMF and to identify genetic markers in linkage with chromosome regions involved in this symbiosis. A tetraploid wheat collection of 127 accessions was genotyped using 35K single-nucleotide polymorphism (SNP) array and inoculated with the AMF species *Funneliformis mosseae* (*F. mosseae*) and *Rhizoglomus irregulare* (*R. irregulare*), and a genome‐wide association study (GWAS) was conducted. Six clusters of genetically related accessions were identified, showing a different mycorrhizal colonization among them. GWAS revealed four significant quantitative trait nucleotides (QTNs) involved in mycorrhizal symbiosis, located on chromosomes 1A, 2A, 2B and 6A. The results of this work enrich future breeding activities aimed at developing new grains on the basis of genetic diversity on low or high susceptibility to mycorrhization, and, possibly, maximizing the symbiotic effects.

## Introduction

Durum wheat (*Triticum turgidum* L. subsp. *durum* [Desf.] Husn.) is one of the most ancient domesticated grain crops^1^ and the only tetraploid wheat subspecies of economic importance. It is widely cultivated around the Mediterranean basin, where it may represent even up to 70% of all wheat acreage, as, for instance, in Algeria and Italy^2^. In such a region, durum wheat is often grown in stressful environmental conditions, with hot and arid environments, where tolerance and resilience to harsh conditions is strongly required. During the *Green Revolution*, breeders imposed a strong selection on durum cultivars based on commercial purposes: local landraces were almost completely replaced by improved semi-dwarf cultivars which showed common characteristics like reduced height and leaf area, limited sprouting and shorter crop cycle^3^. However, such an effort, aimed at improving wheat yield and grain quality, may have resulted in a loss of genetic variability between accessions^4^, and decreased resistance to stress^5^.

The climate crisis, characterized by rising temperatures and water deficient conditions, and the need for a more sustainable agriculture to mitigate the environmental pollution and meet the increasing demand for food^6^, is prompting scientists to recognize the importance of new sources of genetic material^7,8^, and of the plant–microbe interactions to increase crop production^9,10^.

Arbuscular mycorrhizal fungi (AMF) are naturally present in different types of habitats^11^, forming associations with the roots of about 80% of all plant species^12^. These associations are mutualistic symbioses where the heterotrophic fungus receives carbon in the form of organic molecules produced by the plant, which in turn obtains mineral nutrients and water absorbed by the fungus^13^. In addition, many studies showed the beneficial role of AMF in improving tolerance and resilience of plants from abiotic and biotic stresses, revealing the great potentiality of these fungi in sustainable and organic agriculture^14,15^.

While there is argument as to whether breeding for enhancing plant mycorrhizal interactions^16–18^, both mycorrhizal colonization and growth may widely vary among plant accessions, and these traits are considered under genetic control^19–21^, although a strong environmental effect and a low heritability have been observed^22,23^.

Genome-Wide Association studies (GWAS) are these days widely used in plant research to detect Quantitative Trait Loci (QTLs) associated with complex traits such as resistance to biotic stresses^24,25^ and yield quality^26,27^. Concerning wheat, QTL mapping was recently applied to marker-assisted selection (MAS) programs for individual traits contributing to yield enhancement^28,29^ and disease resistance^30,31^. Despite the current relevance of the topic, very few studies related to the detection of genetic markers in linkage with chromosome regions involved in AM colonization through GWAS are available^19,32^.

In durum wheat, a large variability of mycorrhizal response has been observed among cultivars^33–35^, then suggesting that scope exists for optimization of plant interactions with AM fungi. Therefore, a thorough knowledge of the genetic material potentially utilizable for breeding, such as landraces and other naked tetraploid species, is needed to avoid risk of reducing mycorrhizal compatibility of new lines.

Here, we analyzed the genetic diversity of 127 accessions belonging to different *T. turgidum* subspecies in relation to their root colonization by two AM fungi (*Funneliformis mosseae* and *Rhizoglomus irregulare*). After a genetic characterization through single-nucleotide polymorphisms (SNPs) markers, a GWAS was implemented to detect Quantitative Trait Nucleotides (QTNs) associated to mycorrhizal susceptibility.

## Results

### Population structure and genetic diversity of the tetraploid wheat accessions

**Figure 1:**
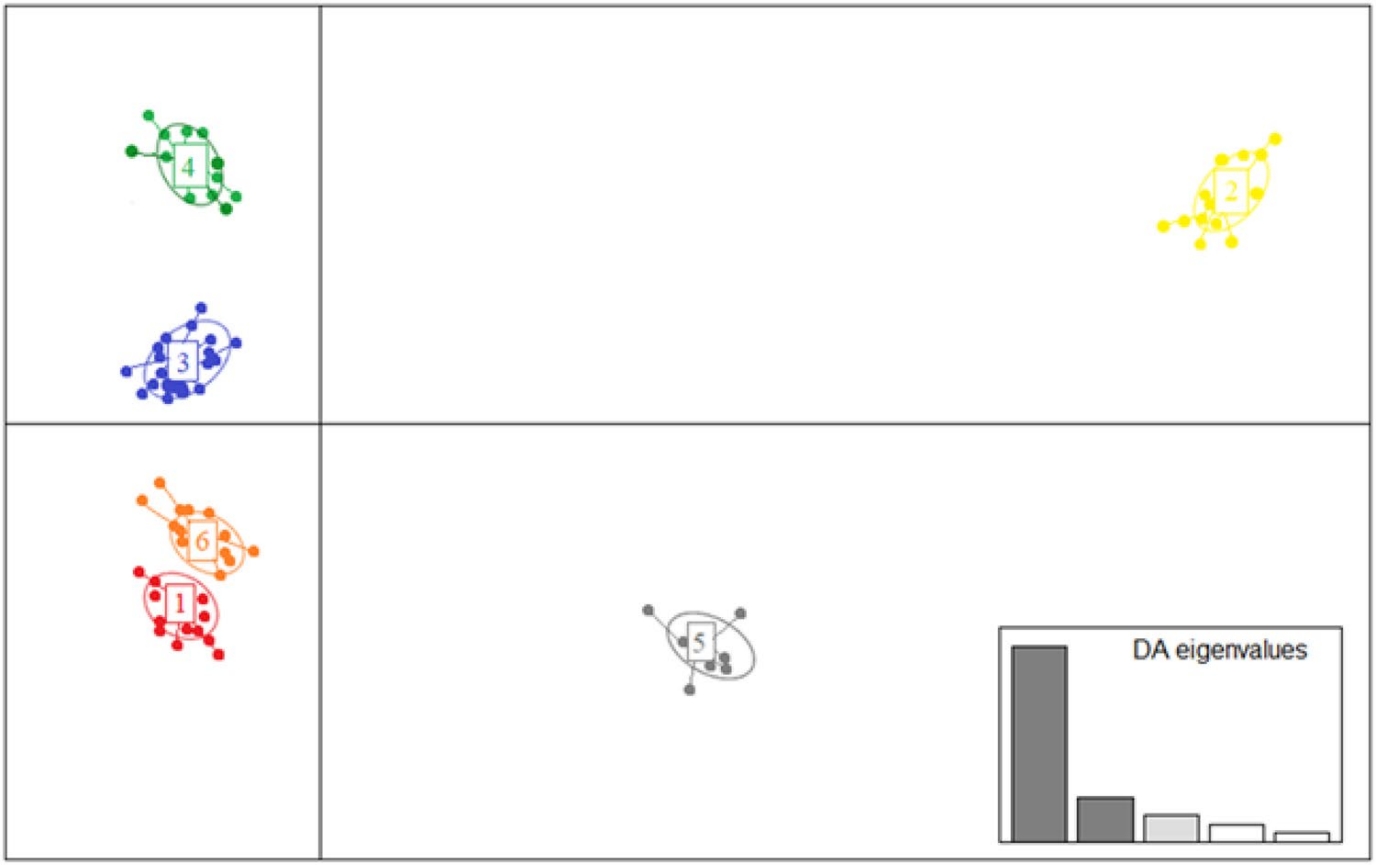
Discriminant analysis of principal components (DAPC) for 127 accessions of *Triticum turgidum* L. used for the analysis. The axes represent the first two Linear Discriminants (LD). Each circle represents a cluster and each dot represents an individual. Numbers represent the different subpopulations identified by DAPC analysis.

After SNP dataset filtering, 21,051 SNP markers were identified and used in the statistical analysis for the evaluation of genetic diversity of the 127 tetraploid wheat accessions. The population structure analyzed by the DAPC method identified 6 clusters of genetically related individuals (Fig. 1). The first 100 PCs and three discriminant eigenvalues were retained. The number of detected groups on which DAPC was carried out was established in coincidence with the lowest BIC value using *find.clusters* function (Fig. S1). In this way, the most markedly different alleles among the 6 groups were used as linear discriminants to perform clusters separations, maximizing the variance between groups and minimizing the variance within groups.

**Figure 2:**
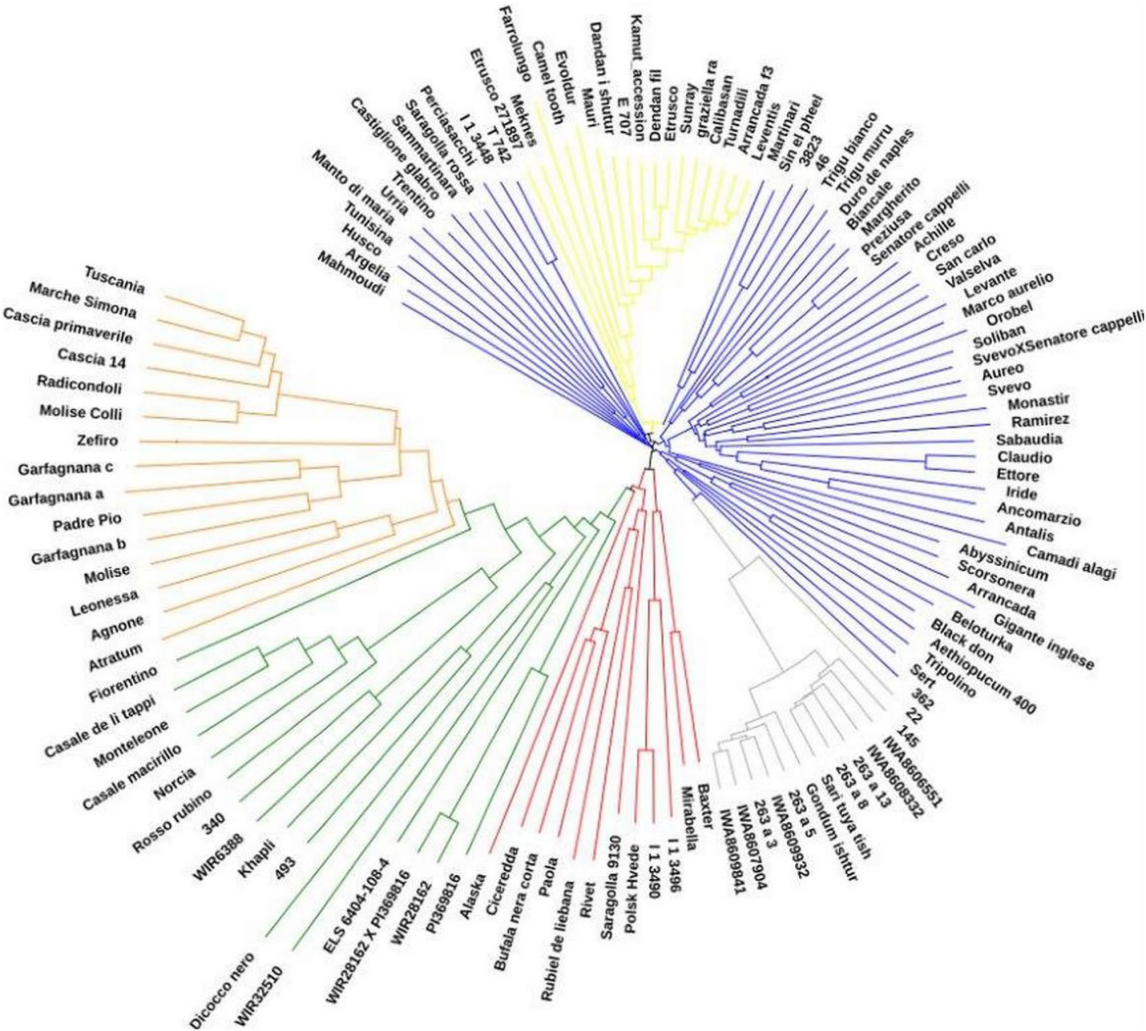
Unrooted tree of 127 tetraploid wheat accessions based on single nucleotide polymorphisms (SNPs) markers genetic distance and colored according to discriminant analysis of principal components (DAPC) clusters. Each tree branches color corresponds to the cluster to which the accession belongs: red for cluster 1, yellow for cluster 2, blue for cluster 3, green for cluster 4, grey for cluster 5 and orange for cluster 6.

The phylogenetic tree, constructed with the Neighbor-Joining algorithm application to a matrix of pairwise distances, better detailed the kinship between accessions and identified clusters that are mainly in agreement with the six subpopulations given by DAPC (Fig. 2). Clusters 1 (red), 2 (yellow), 4 (green), 5 (grey) and 6 (orange) found with DAPC were well identified within the phylogenetic tree. Cluster 3 (blue) was divided into four principal branches which identified four subpopulations, three of them were closer and the fourth more distant.

Groups identified by DAPC, only partially matched the morphological affiliation of the accessions: however, most of ssp. *durum* accessions (42 out of 47) grouped in cluster 3, most (27) of ssp. *dicoccum* accessions grouped in clusters 6 and 4, and most (26) of ssp. *turanicum* accessions in clusters 2 and 5. Some accessions identified as ssp. *polonicum* (8 out of 13) clustered with the main group containing ssp. *durum* accessions (cluster 3), while the other (5) accessions were included in cluster 1, which also contained some ssp*. durum* accessions, along with three accessions described as ssp. *turgidum.* Interestingly, the 3 ssp. *palaeocolchicum* accessions and one of ssp. *carthlicum* (PI 341800) grouped in cluster 4, with ssp. *dicoccum* accessions*.* Another accession described as ssp*. carthlicum* (PI 70738) clustered with ssp. *turanicum* accessions of cluster 5. A single accession of ssp. *dicoccon* (Farrolungo) was included in cluster 2 with different ssp. *turanicum* accessions.

AMOVA results for K = 6 showed a high variability within accessions (91.67% of the total variance) but confirmed a significant difference between clusters (p-value = 4.99975e−05) to which 8.33% of the total variance is attributed. In the wake of such results, subsequent analyses were performed following these genetically determined clusters.

### Mycorrhizal status and colonization level of wheat accessions

The typical symbiotic structures of AMF, such as hyphae, arbuscules, vesicles and spores were observed under the optical microscope, for all accessions (Fig. S2). Roots colonization levels of plants were strongly dependent on the accession, with a high variability both when inoculated with *F. mosseae*, ranging from a minimum of 12.7% (Ramirez, ssp. *durum* of cluster 3) to a maximum of 84.1% (Sin El Pheel, ssp. *polonicum* of cluster 3), and with *R. irregulare*, ranging from 7.0% (Garfagnana b, ssp. *dicoccum* of cluster 6) to 67.7% (Soliban, ssp. *polonicum* of cluster 3) (Table S1). Actually, a high level of variability occurred within genetic clusters as assessed by ANOVA, which showed a significant effect of the interaction of AM fungus and genetic cluster (Table 1): averaging over each cluster, root colonization by *F. mosseae* was significantly higher in accessions belonging to clusters 1, 2, 3, and 5, (ranging from 44.0 to 50.0%) than in accessions of cluster 4 and 6 (ranging from 25.7 to 33.6%) (Table 2). On the other hand, *R. irregulare* colonization was higher in accessions belonging to clusters 2 and 3 than in clusters 4 and 6. In addition, *R. irregulare* produced a consistently and significantly lower level of colonization than *F. mosseae* in cluster 1, 3, 5, 6, but not in clusters 2 and 4 (Table 2, Fig. S3).

**Table 1:** Nested ANOVA results of the effect of arbuscular mycorrhizal fungal isolates (AMF), genetic cluster of *Triticum turgidum* (cluster), and wheat accession, on the level of host root mycorrhizal colonization.

Source	F-value	P-value
AMF	18.0	0.015
Cluster	13.2	< 0.001
AMF × cluster	3.2	0.01
Accession	6.1	< 0.001

**Table 2:** Percentage of colonized root length of tetraploid wheats (means and standard errors, SE), by arbuscular mycorrhizal fungus (AMF) and genetic clusters.

Cluster	AMF							
	*Funneliformis mosseae*				*Rhizoglomus irregulare*			
	Mean	SE			Mean	SE		
1	44.0	17	C	b	33.0	14.1	BC	a
2	45.3	11.8	C	a	43.6	13.8	DE	a
3	45.0	13.9	C	b	40.0	14.8	CDE	a
4	33.6	10.8	B	a	30.2	13.8	B	a
5	50.0	13.2	C	b	36.6	12	BCD	a
6	25.7	8.3	A	b	17.3	7.6	A	a

Means followed by the same letter do not differ significantly (P < 0.05). Capital letters (in columns) refer to differences among clusters within *Funneliformis mosseae* or *Rhizoglomus irregulare* treatments. Small letters (in rows) refer to differences between AMF within a cluster. Simple effect test with Sidak correction.

The highest root colonization (> 60%) by *F. mosseae* was found in accessions included in clusters 3, 5 and 1, while the highest (> 60%) values of *R.irregulare* colonization were reached in clusters 2, 3 and 5. It is of note that some accessions (marked with an asterisk in Table S1)—like Marco Aurelio, Antalis, Iride, and Creso—are modern cultivars (released after 1970), which, in average, didn’t show a reduction of colonization level in respect of old cultivars and landraces as confirmed by an ANOVA performed on the subset of accessions of ssp. *durum* falling in cluster 3 (P = 0.207 and 0.127 for *F. mosseae* and *R. irregulare*, respectively; n: 40).

### Shoot dry weight

The average dry weight of plants from all accessions of *F. mosseae* and *R. irregulare* treatments was 194.1 g and 192.8 g, respectively, but large differences occurred, spanning from 79.7 g in the cross between WIR28162 and PI 369816 (both ssp. *palaeocolchicum*), to 342.2 g in Manto di Maria (ssp. *durum*), after *R. irregulare* inoculation, and from 86.6 g in Polsk Hvede (ssp. *polonicum*) to 331.3 in Dicocco nero (ssp *dicoccon*), after *F. mosseae* inoculation (Table S1).

ANOVA (Table 3) revealed that a high level of variability on plant shoot dry weight occurred among genetic clusters and among accessions within each cluster. Dry weight was usually lower in accessions belonging to clusters 1 and 6 than in those belonging to cluster 2 and 5, and was affected by AM fungal isolate in accessions belonging to clusters 2, 4 and 6. Actually, inoculation with *F. mosseae* was associated to a higher dry weight of plants in clusters 4 and 6, harboring accessions described as ssp. *dicoccum*, while the opposite trend was observed in accessions belonging to cluster 2 (Table 4).

**Table 3:** Nested ANOVA results of the effect of arbuscular mycorrhizal fungal isolates (AMF), *Triticum turgidum* clusters and accession, on plant shoot dry weight.

Effect	F-value	P-value
AMF	0.02	0.889
Cluster	3.79	0.023
AMF × cluster	6.83	< 0.001
Accession	5.69	< 0.001

**Table 4:** **S**hoot dry weight of tetraploid wheat plants (means and standard errors, SE), by arbuscular mycorrhizal fungus (AMF) and genetic clusters.

Cluster	AMF							
	*Funneliformis mosseae*				*Rhizoglomus irregulare*			
	Mean	SE			Mean	SE		
1	170.07	8.26	A	a	167.88	9.71	B	a
2	210.97	7.18	BC	a	233.63	8.87	D	b
3	194.99	4.00	B	a	202.66	4.93	C	a
4	219.20	10.72	C	b	184.27	9.60	BC	a
5	204.53	7.48	BC	a	206.91	8.57	CD	a
6	155.42	6.05	A	b	130.49	4.22	A	a

Means followed by the same letter do not differ significantly (P < 0.05). Capital letters (in columns) refer to differences among clusters within *Funneliformis mosseae* or *Rhizoglomus irregulare* treatments. Small letters (in rows) refer to differences between AMF within a cluster. Simple effect test with Sidak correction.

Interestingly, in the remaining clusters, some accessions, such as Saragolla (cluster 1), T-742, Ettore and Iride (cluster 3) and IWA8608332 (cluster 5) showed a larger dry weight when inoculated with *F. mosseae*. On the contrary, accession 46 (PI 254215) (cluster 3) showed the opposite trend.

At the cluster level, there was a low correlation (Pearson’s r < 0.5) between plant dry weight and root colonization, with the exception of cluster 2, where a significant (P < 0.001), although moderate correlation was detected (Pearson’s r = 0.505, with *F. mosseae*; r = 0.518 with *R. irregulare*).

### Association mapping and gene identification for AMF colonization

The GWAS analysis identified four unique QTNs for *F. mosseae* and *R. irregulare* (Figs. S4, S5). One QTN located on chromosome 1A (QTLamf-1A) was explaining 12.9% of the variance of *F. mosseae* colonization. The colonization of *R. irregulare* was associated with three MTAs located on chromosomes 2A (QTLamf-2A), 2B (QTLamf-2B) and 6A (QTLamf-6A), explaining 10.5% to 15.0% of the phenotypic variance (Table 5).

**Table 5:** Significant marker trait associations (MTAs) and corresponding quantitative trait loci (QTLs) for root colonization of tetraploid wheats (*Triticum turgidum*) by arbuscular mycorrhizal fungi.

Fungus	QTLs	Marker	Chr	Physical pos. (Mb)	P-value FarmCPU model	R^2^ (%)
*R. irregulare*	QTLamf-2A	AX-94536561	2AL	605.18	2.519E−06	10.49
	QTLamf-2B	AX-95019471	2BL	693.73	3.297E−06	13.38
	QTLamf-6A	AX-94438966	6AS	135.24	5.424E−06	14.96
*F.mosseae*	QTLamf-1A	AX-94839893	1AS	Unknown	7.182E−07	12.91

Chr., chromosome; Physical Pos., position on the chromosome in Mb; R^2^, variance explained by marker in percentage (%).

172 genes located close to these significant markers were identified and the relevant protein domains were annotated (Table S2). The interval to search for candidate genes was set to ± 300 Mb from previous literature^35^.

## Discussion

DAPC analysis based on the 21,051 SNPs provided a global picture of genetic relationship and population structure, also confirmed by AMOVA analysis, of the 127 accessions of different subspecies of tetraploid wheats which grouped in 6 clusters. These clusters only partially matched with the morphological taxonomy of the selected varieties and landraces used in this work, highlighting the occurrence of high genetic variability between accessions belonging to the same subspecies.

*Triticum turgidum* ssp. *durum* accessions, mainly gathered in cluster 3, were, however, divided into 4 main groups easily identified within the genetic tree. The first two groups, formed by ancestral accessions with different geographical origin (Italy, Tunisia, Ecuador, Hungary and Germany), and old Sicilian durum wheats, respectively, were separated by a cluster of *T. turgidum* spp. *turanicum* accessions with little genetic variability and by a cluster of *T. turgidum* ssp. *polonicum* accessions. This result is of particular interest since such genetic proximity could be linked to crosses between *durum* and *turanicum* subspecies which originated *T. turgidum* spp. *polonicum* accessions. In addition, within cluster 3, a low genetic variability was showed by modern varieties of *T. turgidum* ssp. *durum* that, unlike the old varieties and landraces, represented a single cluster within the tree. This distribution reflects some results already obtained in previous works which confirmed how the strong selection by breeders -aimed at the constitution of very productive varieties- has considerably reduced the genetic variability between accessions^36,37^. On the contrary, strong genetic differences were highlighted within the second cluster of *T. turgidum* ssp. *turanicum* accessions (cluster 5) characterized by the Iranian origin for the majority of the accessions included. Due to the scarce number of accessions belonging to the ssp. *carthlicum*, *turgidum* and *paleocolchicum*, accurate explanations of analysis results were not possible. Though, the two *T. turgidum* ssp. *paleocolchicum* accessions (WIR28162 and PI_369816) and their cross were located within the same DAPC cluster and on very close branches, at odds with the two *T. turgidum* ssp. *carthlicum* accessions (WIR32510 and 22), whose higher genetic diversity is probably connected to a different origin (Russia and Iraq, respectively). *T. turgidum* ssp. *dicoccon*, assigned to two clusters by the DAPC analysis, identified a clear group within the genetic tree but showed distinctive molecular traits which highlighted a significant genetic diversity already confirmed in previous works^38,39^. Finally, genetic variability was also assessed at accession level: Ciceredda, Paola and Bufala nera corta, defined as the same accession from previous literature^40^, were close to each other but constituted different branches within the tree.

Our results support the role of the genetic variation of the plant host in determining roots colonization by AMF^19,35,41^, as well as the role of the fungal symbiont. In addition, our data hold the findings that modern durum wheat varieties are not affected in their capacities to get colonized by AM fungi, often presenting, on the contrary, high levels of mycorrhizal susceptibility^42,43^. Although the breeding processes which led to the establishment of new lines apparently did not impact those chromosomal traits decisive for the association between roots and fungi^35^, here we found some accessions, such as Ramirez and Ettore, that showed a lower level of colonization in comparison with landraces.

Certainly, the use of genetically based clusters identified by DAPC analysis revealed different susceptibility to AMF colonization. On average, *F. mosseae* showed higher root colonization compared to *R. irregulare*, confirming recent results of Mustafa et al. study^43^, where the same fungi were used to inoculate wheat and the highest level of mycorrhizal colonization was reached by *F. mosseae*. Conversely, this observation contradicts De Vita et al. research^35^, where wheat roots appeared to be most colonized *R. irregulare*. This inconsistency may be due to the different selection of wheat accessions, though environmental factors such as temperature and light intensity, which have been shown to be involved in determining root colonization^44–47^ may have contributed to enhance the mycorrhizal colonization levels.

In addition to light and temperature, other factors related to the host plant, such as root morphology, root to shoot ratio and exudate production, may affect AMF-host interactions^48–50^. In particular, second-order roots typically show the highest mycorrhizal colonization, while first-order roots usually present a lower rate of mycorrhizal symbiosis, principally due to their younger age^51^. Plant exudates, mainly composed by carbohydrates, carboxylic acids and amino acids, represent a convenient source of carbon and energy, mediating the microbe growing in the rhizosphere^52^. Finally, root/shoot ratio seems to be inversely correlated with mycorrhizal dependency^49^. In turn, many studies showed how mycorrhizal colonization had a marked impact on these factors, enhancing lateral root formation and branching^53,54^, modulating metabolite profiling of root exudates^55^ and affecting the root to shoot ratio^56^ in the host plant.

Independently on the differences between the two AMF isolates, the high variability in root colonization is confirmed in all wheat subspecies tested, suggesting that such a trait should be considered in breeding programs. Although a low correlation between colonization and plant weight was found in this experiment, it seems possible to breed for maximizing the root colonization, which can help in maintaining AMF populations in soil, avoexudding undesirable effects on plant growth.

Earlier research works identified genetic traits in wheat associated with mycorrhizal colonization. Two studies on *T. aestivum* discovered a positive effect of chromosomes 1A, 5B, 6B, 7B, 5D, and 7D for mycorrhizal response^19^ and six QTL regions involved in wheat-AMF associations on chromosomes 3A, 4A and 7A^32^. Recently, seven putative QTLs were linked with durum wheat mycorrhizal susceptibility, and were located on chromosomes 1A, 2B, 5A, 6A, 7A and 7B, were detected^35^. These studies however did not consider the allele pool of wheat subspecies that may contribute with novel variation controlling AMF colonization.

In this study, associations on chromosomes 1A, 2B and 6A were in common with that reported in De Vita et al. study^35^ on durum wheat. Nevertheless, QTLs positions identified by this last research were expressed in cM and the approximate conversion to Mb would need further studies to compare their positions with our QTNs. The use of a stringent Bonferroni correction lowered the number of QTN identified because was intended to protect from Type II errors^57^ while supporting the relevance of the discussed QTNs. The associations surpassing the threshold co-localized to many traits significantly associated with quantitative phenotypic data—grain yield, biocontrol of *Fusarium*, roots colour—which appear to be affected by AMF-plant interaction^58–60^. Close to QTLamf-6A, QTLs for epistatic effects for flour color traits (QFb.cerz-6AL.2)^61^, and for grain length (qgl6A) and weight (qtkw6A)^62^ were detected. QTLamf-2A co-localize to QSPS-2A.4^63^ and to the markers DArT3154, DArT3155 and DArT3156, significantly associated to yield-related trait in wheat^64^. The microsatellite Xgwm120 and the SNP 1072874, significantly and respectively associated with QTL for scab^65^ and Fusarium head blight (FHB)^66^ resistance, were detected in the same chromosome region AX-95019471. Finally, on chromosome 1A, where QTLamf-1A for *F. mosseae* colonization was identified, a QTL for grain weight (QGw1.ccsu-1A)^62^, and a QTL for Fusarium head blight resistance (QFhs.nau-1AS)^66^ were previously observed.

172 functional genes were mapped within ± 300 Mb interval from the identified QTLs and many of them may be related to mycorrhizal colonization. Genes involved in activities which seemed to be increased during the establishment of mycorrhizal symbiosis are close to QTLamf-2B, such as the expression of root plasma membrane ATPase^67,68^, the oxidation–reduction processes, both in roots and in leaves^69^, and the root conversion of sugars into lipids and their translocation to the extraradical mycelium^70,71^. Root-expressed functional genes for hydrolytic enzymes, organic and inorganic N transport, ATP binding protein kinase activities and glycoside transport, associated by recent researches to AMF root colonization^72–76^ were located close to QTLamf-2A. Genes related to carbohydrate metabolic process (carbohydrate metabolism and synthesis of cell wall polysaccharide precursors) and Calmodulin binding proteins codification, whose expression resulted higher in AMF colonized plants^41,77^ were found close to QTLamf-6A.

This work, which analyzed several accessions of tetraploid wheat belonging to *T. turgidum* ssp. *turgidum*, ssp. *paleocolchicum*, ssp. *carthlicum* and ssp. *polonicum*, in addition to a large collection of other previously not tested *T. turgidum* ssp. *durum* and spp. *dicoccum*, made it possible to identify four QTN possibly contributing to mycorrhizal susceptibility. These results could enrich future breeding activities aimed at developing new grains on the basis of genetic diversity on low or high susceptibility to mycorrhization, and, possibly, maximizing the symbiotic effects.

## Materials and methods

### Plant material and genetic structure analysis

Plant material is part of a tetraploid wheat (*Triticum turgidum*, 2n = 4× = 28; AABB genome) collection at the University of Florence, Italy (Table S1). *T. turgidum* accessions belonging to eight different subspecies (ssp.) were selected among those with the highest genetic diversity in a larger collection: 2 belonging to ssp. *carthlicum*, 28 to ssp. *dicoccon*, 47 to ssp. *durum*, 3 to ssp. *paleocolchicum*, 13 to ssp. *polonicum*, 31 to ssp. *turanicum* and 3 to ssp. *turgidum*. They were genotyped by 35k wheat breeders’ Axiom array using Affymetrix GeneTitan system at Bristol Genomic Facility (England).

Genomic DNA of 127 accessions was extracted from leaf tissue of each genotype using a standard cetyltrimethylammonium bromide (CTAB) protocol^78^ and successively treated with RNase-A (New England Biolabs UK Ltd., Hitchin, UK) according to the manufacturer’s instructions. DNA was checked for quality and quantity by electrophoresis on 1% agarose gel and Qubit fluorimetric assay (Thermofisher), respectively. The Axiom Wheat breed Genotyping Array 35K was used to genotype the 127 samples for 35.143 SNPs using the Affymetrix GeneTitan system at Bristol Genomics Facility according to the procedure described by Affymetrix (Axiom 2.0 Assay Manual Workflow User Guide Rev3). The array was previously developed for the three genome AABBDD of *Triticum aestivum* and for this reason, working on *T. turgidum*, which lacks D genome, a variant call rate threshold of 92% was used instead of the default value (97%) to account for the lower call rates obtained from tetraploid wheat. Using custom command presented in the Axiom Analysis software, each accession was taken into consideration separately for calculating the number of monomorphic and polymorphic SNP markers, the heterozygosity level and the types of nucleotide substitution. Monomorphic SNP markers and those with missing data points were excluded from analysis. SNP markers were filtered for minimum allele frequency (MAF) greater than 1% and failure rate lower than 20%, starting from a total of 21,051 SNPs.

In order to provide an efficient description of accessions genetic clusters using a few synthetic variables, a discriminant analysis of principal components (DAPC) was implemented, using R software v.3.6. (R core team, 2013) with R\adegenet package^79^ and R\poppr package^80^. DAPC first step was the data transformation using principal component analysis (PCA) while the second step was the discriminant analysis performing on the retained principal components (PCs). Groups were identified using k-means, a clustering algorithm which finds a given number (k) of groups maximizing the variation between them. To identify the optimal number of clusters, k-means was run sequentially with increasing values of k, and different clustering solutions were compared using Bayesian Information Criterion (BIC). The optimal clustering solution should represent to the lowest BIC^81^.

Subsequently, starting from a matrix of pairwise distances estimated by using Maximum Composite Likelihood (MCL) model, an unrooted, Bayesian tree was obtained by applying the Neighbor-Join algorithms^82^ in R software with R/ape 3.1^83^. The branches of the tree corresponding to each genotype were differently colored according to the cluster (found with the DAPC) they belong to. A Bayesian tree and DAPC analysis allowed to investigate levels and patterns of genetic diversity among the examined accessions, utilized to assess the association between the wheat accessions and their root mycorrhizal colonization.

With the purpose of detecting the genetic variation within population and supporting the population structure obtained with DAPC, analysis of molecular variance (AMOVA) was performed for K = 6 subdivision levels using R\poppr package^80^.

### Fungal material

Fungi used in the experiment were: *Funneliformis mosseae* (T.H. Nicolson & Gerd.) C. Walker & A. Schüßler, isolate IMA1 and *Rhizoglomus irregulare* (Błaszk., Wubet, Renker & Buscot) Sieverd., G.A. Silva & Oehl, isolate IMA6. Isolates were obtained from pot-cultures maintained in the collection of the Microbiology Laboratories of the Department of Agriculture, Food and Environment, University of Pisa, Italy. The fungal inocula were produced in greenhouse on *Trifolium alexandrinum* L. as host plant, grown for 6 months in a mixture (1:1 by volume) of sterilized soil and calcinated clay (OILDRI Chicago, IL, USA). At harvest, roots were cut in ca. 1-cm fragments and mixed with the substrate to form a homogenous crude inoculum mixture, to be used for wheat inoculation. Before starting the experiment, biological activity of such inocula was assessed by using the Mycorrhizal Inoculum Potential (MIP) bioassay, as described in^84^.

### Experimental setup

The experiment examined 127 accessions of *T. turgidum*. Seeds were sown in 6 cell (45 × 55 mm) plug trays filled each with 100 mL of a mixture of *F. mosseae* or *R. irregulare* crude inoculum and sterilized calcined attapulgite clay, 1:1 by volume. Three replicate cells for each tray were utilized for each accession.

Plants were grown in an unheated greenhouse and watered with tap water until 10 days after planting (DAP). Then, nutrients were supplied by adding 3 mL of a low P nutrient solution twice a week to each cell, up to the end of the experiment. The nutrient solution contained NH_4_NO_3_ (40 mg/L), KNO_3_ (101 mg/L), KH_2_PO_4_ (0.5 mg/L), K_2_SO_4_ (14 mg/L), KCl (11 mg/L) from commercial grade compounds.

Throughout the growing period, from April 2019 to early June 2019, daily minimum and maximum outdoor temperatures were in the range of 5.8–17.2 °C and 12.7–25.5 °C, respectively. Every week, trays were randomly moved to avoid position effects.

At harvest, seventy days after emergence, plants were removed, and shoot separated from roots. Shoots were dried in an oven at 50 °C for 72 h and weighted, roots were stored for later analysis of mycorrhizal colonization.

### Mycorrhizal quantification

Plants roots were cleared with 10% KOH in a water bath (80 °C for 15 min) and stained with Trypan blue in lactic acid (0.05%) after 10 min in 2% aqueous HCl. A dissecting microscope (Wild, Leica, Milano, Italy) at 25× or 40× magnification was used in order to estimate percentages of AMF colonization by the gridline intersect method^85^. Mycorrhizal colonization on roots samples was also assessed and observed under a Polyvar light microscope (Reichert-Jung, Vienna, Austria) at 125× and 500× magnification.

### Phenotypic data analyses

Data of shoot dry weight and root colonization from the wheat accessions were analyzed in SPSS v. 25 using a general linear model including AMF as fixed effect, accession and cluster as random effect and the interaction between AMF and cluster.

Normality and heteroskedasticity were checked and transformations were not required for dry weight. Root colonization data were analyzed after square root transformation. Simple regressions were undertaken on the mean for each accession.

### Genome wide association analysis

GWAS was carried out using 127 tetraploid wheat accessions phenotyped for *F. mosseae* and *R. irregulare* fungal colonization. The GWAS was performed using the R\mvp package^86^, using the association model of fixed and random model Circulating Probability Unification (FarmCPU)^87^. Principal components calculated on SNPs diversity were included as covariates to capture the population structure existing in the panel. A kinship matrix was calculated on the same SNP set and included as random factor to the model to account to the relatedness among individuals. A multiple test correction was applied with a Bonferroni threshold for a nominal test p value of 0.1. QTNs notation was done for markers surpassing the Bonferroni threshold while allelic effects and phenotypic variance explained by each marker were derived from the model. Genetic position of QTNs was derived from the CerealsDB database (cerealsdb.uk.net) of Bristol University in order to compare them to QTL already reported in literature. Candidate genes were identified searching gene models within a fixed interval of ± 300 Mb around the corresponding marker position on the Ensembl plant's database (https://plants.ensembl.org). The Pfam 32.0 database was queried to derive the protein domain and the possible function candidate genes.

### Ethical statement

Experimental research and field studies on plants (either cultivated or wild), including the collection of plant material, comply with relevant institutional, national, and international guidelines and legislation.

## Supplementary Information

Supplementary Information 1.

Supplementary Information 2.

Supplementary Information 3.
