# Supplementary material for: Genetic variability assessment of 127 Triticum turgidum L. accessions for mycorrhizal susceptibility-related traits detection
Source: Sci Rep. 2021 Jun 28;11:13426. doi: 10.1038/s41598-021-92837-1 (PMC8239029; doi:10.1038/s41598-021-92837-1)
Supplement: Supplementary file 1 — Supplementary Information 1. [file 41598_2021_92837_MOESM1_ESM.docx]

**Scientific reports Supporting Information**

**Article title:** Genetic variability assessment of 127 Triticum turgidum L. accessions for mycorrhizal susceptibility-related traits detection

**Authors:** Paola Ganugi, Alberto Masoni, Cristiana Sbrana, Matteo Dell’Acqua, Giacomo Pietramellara, Stefano Benedettelli, Luciano Avio

**The following Supporting Information is available for this article:**

**Fig. S1. Statistical determination of the optimum number of clusters by discriminant analysis of principal components (DAPC).** The elbow in the curve matches the smallest BIC, and clearly indicates 6 clusters should be retained.

**Fig. S2. Arbuscular mycorrhizal colonization of *Funneliformis mosseae* (FM) in Aureo (*Triticum turgidum* L.) root.** (a) Intraradical vesicles and intraradical spores. (b) Arbuscular coil. (c) hyphal coils.

**Fig. S3. Frequency distribution of means for *Funneliformis mosseae* (FM) or *Rhizoglomus irregulare* (RI) in 127 tetraploid wheat accessions at 70 days after emergence.**

**Figure S4. Manhattan plot (−log10[P]) genome-wide association plot) of a genome-wide association study on 127 tetraploid wheat accessions colonized by *Funneliformis mosseae* (a) and *Rhizoglomus irregulare* (b).** The genome-wide significance level is set at 5.462E-06 and plotted as the dotted line.

**Figure S5. Quantile-quantile (QQ) plot.** Q-Q plots display the observed association P-value for all SNPs on the y-axis versus the expected uniform distribution of Pvalues under the null hypothesis of no association on the x-axis. The strongly associated SNPs deviate from the diagonal at the upper-right end of the plot.

**Table S1. List of accessions of *Triticum turgidum* subspecies included in the experiment and phenotypic data related to AMF colonization and dry weight.**

**Table S2. Candidate genes identified for all the sequences of the markers mapped in the regions of QTNs.**


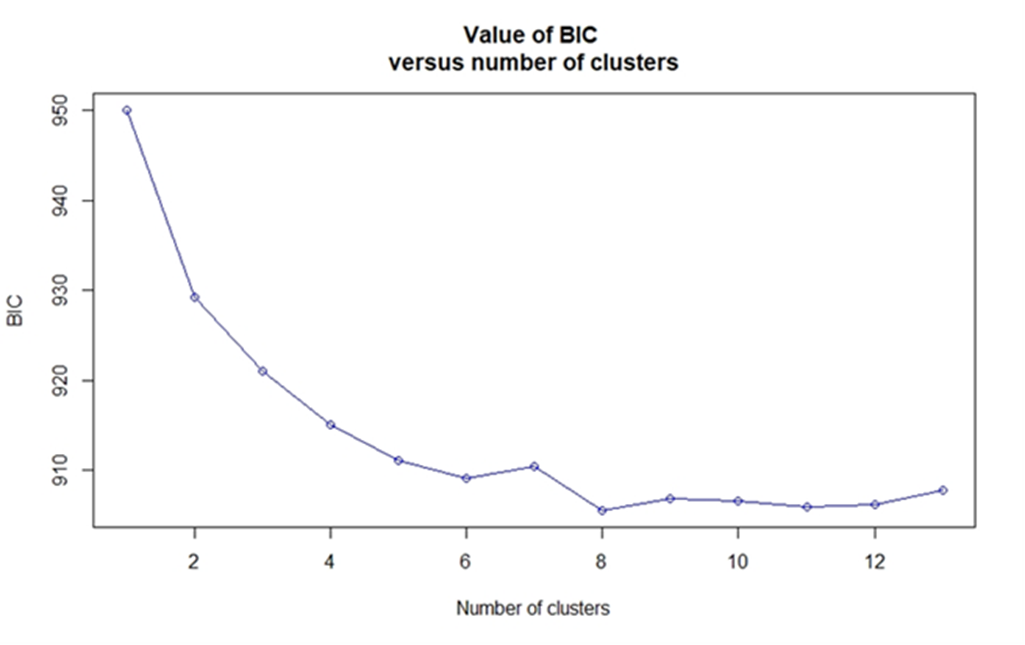


**Fig. S1**

**
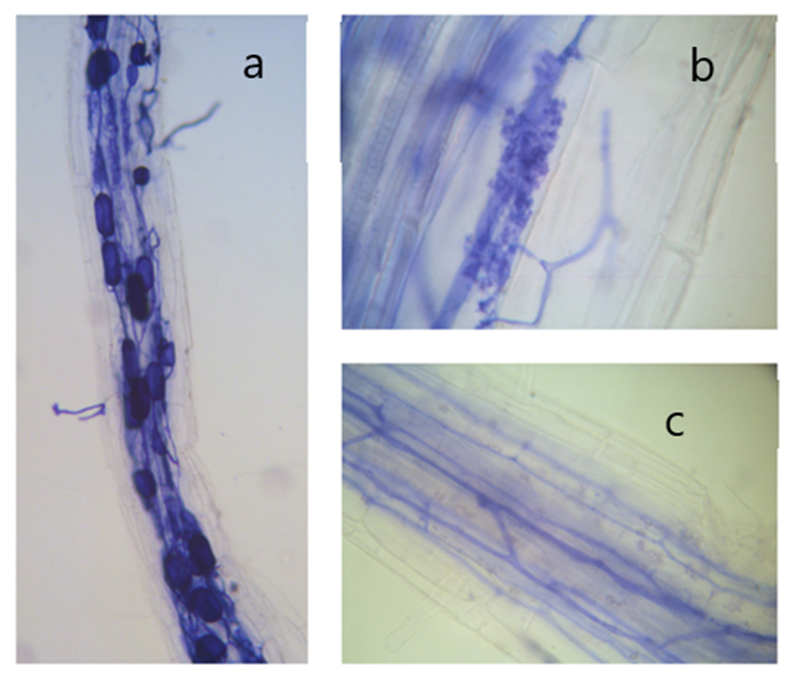
**

**Fig. S2**

**
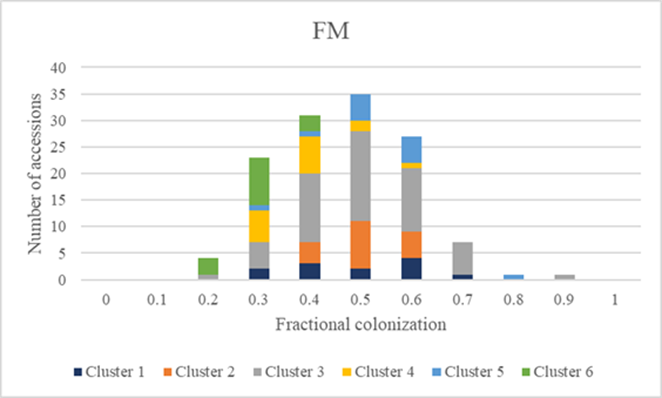
**

**
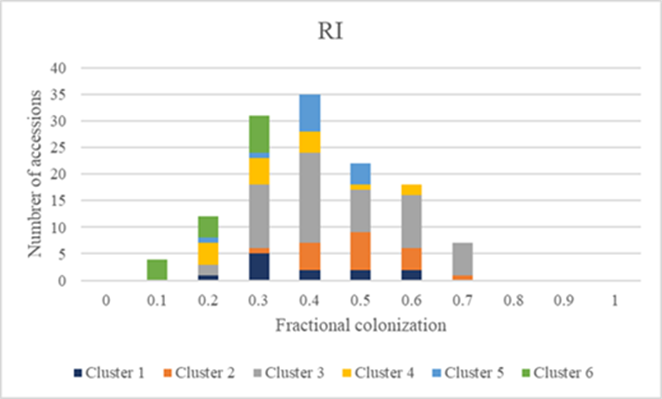
**

**Fig. S3**

**
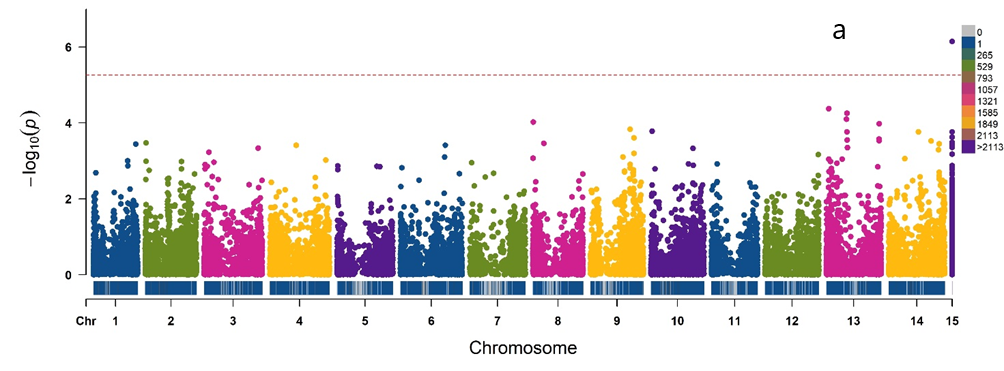
**

**
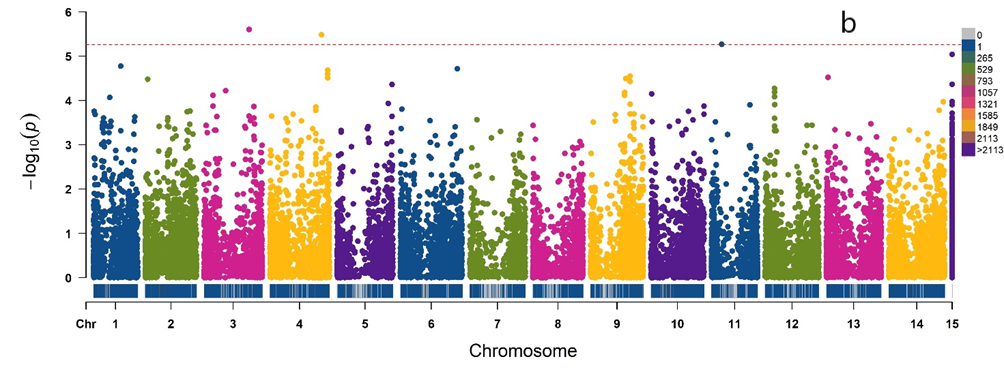
**

**Fig. S4**

**
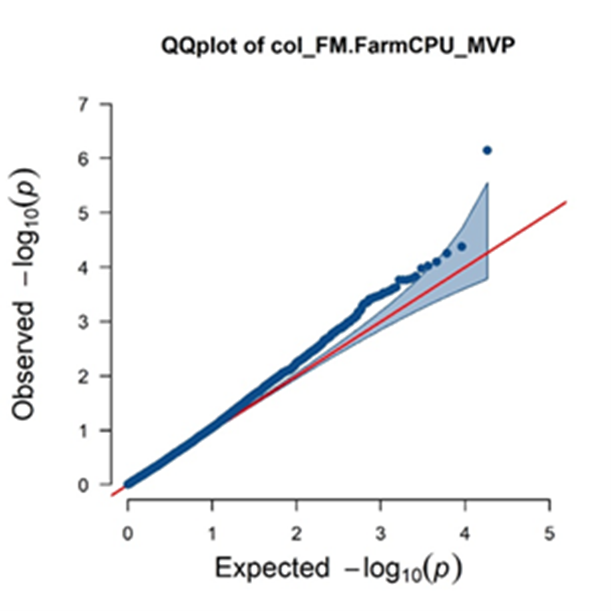
**

**
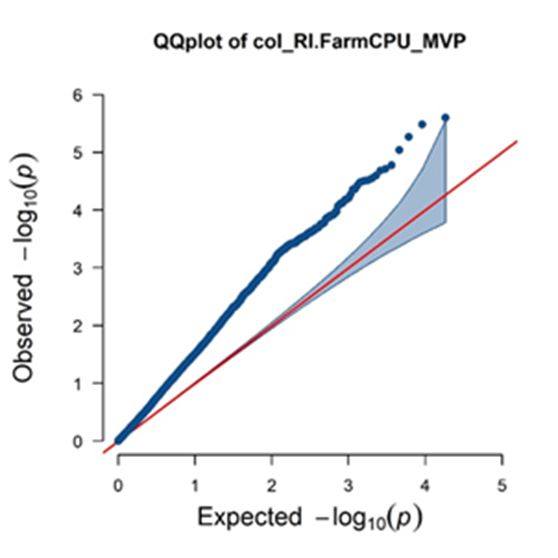
**

**Fig. S5**
